# Reply to the letter regarding the article ‘Sodium–glucose cotransporter 2 inhibitors influence skeletal muscle pathology in patients with heart failure and reduced ejection fraction’

**DOI:** 10.1002/ejhf.3442

**Published:** 2024-09-18

**Authors:** Nathanael Wood, Klaus K. Witte, T. Scott Bowen

**Affiliations:** ^1^ School of Biomedical Sciences, Faculty of Biological Sciences University of Leeds Leeds UK; ^2^ Leeds Institute of Cardiovascular and Metabolic Medicine University of Leeds Leeds UK

We are grateful for the important and timely comments of Stöllberger and colleagues about our study on the effects of sodium–glucose cotransporter 2 inhibitors (SGLT2i) on skeletal muscle in people with heart failure and reduced ejection fraction (HFrEF).^1^ We agree entirely that further research is required to understand the effects of these agents on skeletal muscle structure and function, especially in older people.

In response to their comments, one of the unique features of our work is that the sample collection spanned the approval and guideline uptake of these agents in people with HFrEF. This allowed us, albeit in a longitudinal manner, to compare people treated with and without these agents. As reassurance, it is standard practice in our laboratory to keep the (anonymized, coded) samples separate from the clinical data until terminal analysis, which ensures no bias. This approach, the longitudinal sample collection, and the requirement of guideline‐directed medical therapy prior to device therapy, comes with both benefits and challenges since, as highlighted, some baseline characteristics such as age, N‐terminal pro‐B‐type natriuretic peptide (NT‐proBNP) levels and prevalence of atrial fibrillation were numerically higher (but not significantly different) between our groups. However, the standard deviations showed a wide distribution (age 73 ± 9 vs. 66 ± 10 years; NT‐proBNP 4052 ± 4813 vs. 2039 ± 2526 ng/L), highlighting the overlap between groups and, in the absence of any correlation with age, this is unlikely to be a confounding factor. Finally, we combined human with highly‐controlled experiments in a mouse model to independently confirm the favourable effects of SGLT2i on muscle pathology.

Stöllberger and colleagues point out that we did not have a definitive diagnosis of the aetiology of HFrEF in all patients, enquired whether all patients underwent a formal neurology consult, and propose that we describe all types and doses of medications. In response, we would propose that since the aetiology of HFrEF in a patient without angina has little bearing on treatment, invasive coronary angiography or cardiac magnetic resonance in older people with HFrEF is not routine. Moreover, we also do not routinely refer patients with HFrEF to neurology to exclude neuromuscular disease in the absence of symptoms or signs. Finally, we would like to reassure Stöllberger and colleagues that, as required prior to prescription of an implantable device for HFrEF, patients were taking optimally tolerated doses of guideline‐directed medical therapy with no differences in doses across groups.

We agree entirely that the addition of global muscle mass or functional strength assessments would have added valuable insight into the effect of SGLT2i in HFrEF. While a direct muscle biopsy at a single site comes with both advantages and limitations, we would like to point out that volitional strength and imaging assessments do not provide similar critical insights into underlying biological mechanisms. Overall, however, we agree that more work is required to better understand the effects of SGLT2i on sarcopenia, especially as the study cited by Stöllberger and colleagues^2^ highlighted considerable inconsistency and did not include any studies in people with HFrEF.

In summary, we believe our work^1^ presents important information on the effects of SGLT2i on skeletal muscle pathology in a generalizable cohort of people with HFrEF, thereby providing a solid foundation for future work.
